# Evaluation of Whitening Effects and Identification of Potentially Active Compounds Based on Untargeted Metabolomic Analysis in Different Chrysanthemum Cultivar Extracts

**DOI:** 10.3390/antiox13121557

**Published:** 2024-12-18

**Authors:** Fenglan Wang, Huiya Liu, Zifeng Huang, Yangyang Zhang, Yitong Lu, Yiwei Zhou

**Affiliations:** 1 College of Horticulture and Landscape Architecture, Zhongkai University of Agriculture and Engineering, Guangzhou 510225, China; wangfl2002@163.com (F.W.);; 2 Zhonghua Modern Agriculture Research Institute, Huadu District, Guangzhou 510800, China; 3 Dongguan Research Center of Agricultural Sciences, Dongguan 523086, China; 4 Guangdong Provincial Key Laboratory of Ornamental Plant Germplasm Innovation and Utilization, Environmental Horticulture Research Institute, Guangdong Academy of Agricultural Sciences, Guangzhou 510640, China

**Keywords:** chrysanthemum, skin whitening, antioxidant, LC-MS

## Abstract

Chrysanthemum is a traditional Chinese medicinal herb. Chrysanthemum extracts are rich in bioactive compounds; however, there are few reports evaluating the whitening effects of organic chrysanthemum extracts. This study assessed the antioxidant and whitening effects of organic extracts from the petals of five chrysanthemum cultivars from Guangdong, China. Significant differences were observed among the five cultivars across various parameters, including *IC_50_* values for tyrosinase inhibition activity, DPPH and ABTS values, UV absorption, and *SPF* values. Additionally, there were notable variations in total flavonoid, total phenolic, and chlorogenic acid contents. The BJ cultivar extract exhibited strong antioxidant capacity and superior whitening effects, containing the highest levels of total flavonoids, total phenolics, and chlorogenic acid. Correlation analysis indicated a significant relationship between total flavonoid content and *IC_50_* of DPPH, and between chlorogenic acid and both *IC_50_* of ABTS and *SPF*. Untargeted metabolomic analysis of three representative cultivars (BJ, WYHJ, and JSHJ) identified 22 compounds potentially related to antioxidant and whitening effects. Compounds significantly correlated with multiple antioxidant or whitening indicators (*p* < 0.05, *r* > 0.8) included tangeritin, hydroquinone, eupatilin, quercetin 3-(6″-malonyl-glucoside), biochanin A, and cyanidin 3-glucoside. These compounds may play crucial roles in the antioxidant and whitening effects of chrysanthemum extracts. The results highlight the promising antioxidant and whitening properties of chrysanthemum extracts, with certain genotypes, such as BJ, showing potential as superior raw material sources.

## 1. Introduction

Sunscreens are widely used to protect the skin from excessive sun exposure, which can lead to various skin issues such as darkening and diseases [1,2]. Hydroquinone has been extensively used in cosmetic formulations as an effective whitening agent due to its ability to inhibit melanocyte metabolism. However, it has been banned due to significant safety concerns. In recent years, sunscreen formulations have continually evolved, extending from the use of single agents inhibiting tyrosinase to complex mixtures [3]. Notably, many natural extracts, such as mulberry, licorice, peony, kiwi, and sophora, have been incorporated into sunscreens for their tyrosinase inhibitory activity, antioxidant properties, or anti-inflammatory effects. However, the range of these natural extracts remains relatively limited, necessitating further development of additional varieties to meet market demands. To date, various natural compounds, including carotenoids, tocopherols, ascorbic acid, flavonoids, and phenolics, have been developed and added to sunscreens [4,5,6,7,8]. Although some natural plant extracts have been found to be applicable in sunscreens, the number of available plant species is currently limited. More natural extracts need to be developed to replace whitening agents such as hydroquinone. Chrysanthemum, in particular, shows significant potential due to its strong antioxidant activity.

The natural extract of chrysanthemum has good potential for application in sunscreens. Chrysanthemum (*Chrysanthemum morifolium* Ramat.) is a perennial herbaceous plant of the Asteraceae family and a traditional Chinese medicinal herb [9]. Chrysanthemum contains numerous bioactive compounds, such as flavonoids [10,11,12], phenolic acids [13,14], lignans [15], triterpenes, and polysaccharides [16], which contribute to its pharmacological effects, including neuroprotection [13], cardiovascular protection [17], anticancer [18,19], and anti-inflammatory activities [11]. Notably, some studies have shown that chrysanthemum extracts possess tyrosinase inhibitory activity, antioxidant, and sunscreen effects. For instance, Chen et al. found that an extract of *C. morifolium* Hang Ju No. 1 obtained using pure water exhibited the highest tyrosinase inhibitory activity, followed by ethanol extracts [20]. Yang et al. evaluated the antioxidant activity of essential oils from three chrysanthemum varieties using DPPH and FRAP assays, finding that all had DPPH radical scavenging activity [21]. Additionally, the content of chrysanthemum in chrysanthemum tea was significantly correlated with antioxidant activity [22]. Gonsalves et al. reported that both ethanol and water extracts of chrysanthemum showed good UV absorption at 295 nm [23]. Some wild chrysanthemum extracts have been found to have excellent antioxidant, antibacterial, and whitening effects, with *C. zawadskii* var. *Lucidum* exhibiting the strongest antioxidant activity among five wild chrysanthemums and *C. makinoi* showing strong in vitro tyrosinase inhibitory activity [24]. These preliminary studies suggest a promising future for developing cosmetics containing chrysanthemum extracts.

Drying is a crucial step in the extraction of natural plant extracts, as it enhances extraction efficiency and facilitates storage and transportation [25]. The drying process is crucial for maintaining the bioactive components of harvested medicinal herbs, as it affects the quality of medicinal products by altering plant chemical composition, active ingredient content, and bioactivity [25,26]. Natural air drying is the most common method, but it is time-consuming, uncontrollable, and results in low drying quality due to environmental exposure [27]. Freeze drying (FD) can enhance the quality of medicinal herbs, with vacuum freeze drying having been proven to preserve the original characteristics of samples to the greatest extent [28,29,30]. It is one of the preservation methods used in the food industry to maintain or improve food quality [31]. Therefore, using vacuum freeze drying for the extraction of chrysanthemum extracts helps preserve chemical components and maintain high quality.

Currently, there are few studies on the whitening effects of natural chrysanthemum extracts. As chrysanthemum is a traditional herbal medicine and its extracts are widely used in various fields, further development of its potential applications in whitening agents has significant advantages, particularly in terms of safety, extensive cultivation, and widespread recognition. This study selected chrysanthemum varieties with medicinal value, widely cultivated in Guangdong, China, as research materials. These include two of the four famous chrysanthemums in China, ‘Gongju’ (GJ) and ‘Boju’ (BJ), which hold significant potential for development as traditional Chinese medicines. Additionally, the study includes two tea varieties, ‘Wuyuanhuangju’ (WYHJ) and ‘Jinsihuangju’ (JSHJ), as well as our own cultivated variety, ‘Yinlianjinhuiju’ (YLJHJ). To preserve the effective components of the chrysanthemum as much as possible, we first pre-treated the samples using vacuum freeze drying. After processing with vacuum freeze drying, ethanol extracts were obtained, and their physiological differences and potential whitening indicators were compared. Three cultivars having significant differences in efficacy were selected for untargeted metabolomic analysis to identify markers potentially associated with whitening efficacy, laying the foundation for developing chrysanthemum-based whitening cosmetics.

## 2. Materials and Methods

### 2.1. Plant Materials

**Figure 1:**
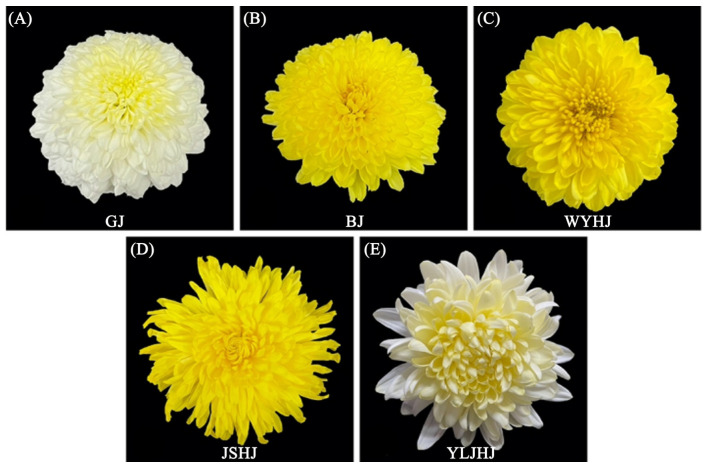
The capitulum inflorescences of five chrysanthemum cultivars from Guangdong, China. (**A**) ‘Gongju’ (GJ); (**B**) ‘Boju’ (BJ); (**C**) ‘Wuyuanhuangju’ (WYHJ); (**D**) ‘Jinsihuangju’ (JSHJ); (**E**) ‘Yinlianjinhuiju’ (YLJHJ).

This study selected chrysanthemum cultivars that are widely cultivated in Guangdong, China, and have certain medicinal value. These include two varieties that are among four famous chrysanthemums in China, ‘Gongju’ (GJ) and ‘Boju’ (BJ), two varieties that can be used for tea, ‘Wuyuanhuangju’ (WYHJ) and ‘Jinsihuangju’ (JSHJ), and our self-bred cultivar, ‘Yinlianjinhuiju’ (YLJHJ). From October 2021 to January 2022, samples of these five cultivars were collected from the chrysanthemum planting base at Guangzhou Huadu District, Zhongkai University of Agriculture and Engineering (Figure 1). All the samples were harvested during peak bloom on sunny days, with the capitulum being the harvested part.

### 2.2. Preparation of Chrysanthemum Extract

Due to the high water content of freshly picked chrysanthemums, a SCIENTZ-10N freeze dryer (Ningbo Xinzhi Biotechnology Co., Ltd., Ningbo, China) was used for vacuum freeze drying to better preserve the active ingredients and quality of the samples. The chrysanthemum samples were pre-frozen in a cold trap at −40 °C for 6 h, followed by 12 h of drying. The dried products were ground into powder using a mortar and pestle after reaching room temperature in a sealed drying container. Then, 0.5 g of coarse powder was accurately weighed and extracted using ultrasound-assisted ethanol extraction with 70% ethanol as the solvent. The powder was then soaked for 20 min with a material-to-liquid ratio of 1:70. Ultrasonic extraction was conducted at 60 °C twice, each for 50 min. The solution was filtered, the residue discarded, and the filtrate combined, adjusting the volume to 100 mL to obtain a chrysanthemum extract with a crude drug concentration of 5 mg/mL. The extract was stored in a refrigerator at 4 °C. The extract was used for tyrosinase inhibitory activity, DPPH radical scavenging activity, ABTS radical scavenging activity, UV absorption and *SPF* analysis, and determination of total flavonoid, total phenolic, and chlorogenic acid contents. All the experiments were performed in triplicate.

### 2.3. Tyrosinase Inhibitory Assay

The tyrosinase inhibitory activity of the chrysanthemum extracts was measured using a modified version of the method described by Kubo et al. [32]. After adding the reaction mixture to the tyrosinase solution and mixing, the samples were incubated at 37 °C for 10 min. L-DOPA solution was then added, mixed, and incubated at 37 °C for another 10 min before transferring to a cuvette. The absorbance of each reaction mixture was measured at 475 nm using phosphate buffer (pH 6.8) as a blank control. The half maximal inhibitory concentration (*IC_50_*) of tyrosine enzyme inhibition rate was estimated through general linear regression.

### 2.4. DPPH Radical Scavenging Activity Assay

The DPPH radical scavenging rate was determined according to the method of Brand-Williams et al. [33]. A 0.06 mmol/L ethanol solution of DPPH radicals was prepared, and its maximum absorption wavelength was determined to be 516 nm. A mixture of 3 mL DPPH solution and 0.1 mL chrysanthemum extract was incubated at room temperature in the dark for 2 h. The absorbance of each reaction mixture was measured at 516 nm using anhydrous ethanol as a blank control. The *IC_50_* of the DPPH radical scavenging rate was estimated through general linear regression.

### 2.5. ABTS Radical Scavenging Activity Assay

The ABTS radical scavenging rate was determined according to a previously described method [34]. A mixture of 0.2 mL 7.4 mmol/L ABTS solution and 0.2 mL 2.6 mmol/L potassium persulfate solution was incubated at room temperature in the dark for 12 h and then diluted with phosphate buffer (pH 7.4) to an absorbance of 0.7 ± 0.02 at 734 nm, forming the ABTS+ stock solution. A mixture of 1 mL chrysanthemum extract and 2 mL ABTS+ working solution was incubated at room temperature in the dark for 10 min. The absorbance of each reaction mixture was measured at 734 nm using phosphate buffer (pH 7.4) as a blank control. The *IC_50_* of the ABTS radical scavenging rate was estimated through general linear regression.

### 2.6. UV Absorption and SPF Analysis

UV absorption spectra were recorded in the range of 200–400 nm using a UV-2600 UV-Vis spectrophotometer. *SPF* was measured in vitro using the spectrophotometer, following the method described by Yang et al. [35]. Chrysanthemum extracts were diluted to a concentration of 0.5 mg/mL with 70% ethanol, and 70% ethanol was used as a blank. The absorbance spectrum was recorded every 5 nm from 290 to 320 nm. The *SPF* value was determined through cumulative calculations. Each point was measured three times, and the Mansur equation [36] was applied:

*SPF* = *CF* × ∑*EE*(*λ*) × *I*(*λ*) × *ABS*(*λ*)

where (*EE*(*λ*)) is the erythema effect spectrum, (*I*(*λ*)) is the solar intensity spectrum, (*ABS*(*λ*)) is the absorbance of the test material, and (*CF*) is the correction factor (−10). Constants are shown in Table 1, as previously described [36].

### 2.7. Determination of Total Flavonoid, Total Phenolic, and Chlorogenic Acid Contents

The flavonoid content of the chrysanthemum extracts was determined using the NaNO_2_-Al(NO_3_)_3_-NaOH colorimetric method [37], with rutin as the standard. The total phenolic content was measured using the Folin–Ciocalteu method [38], with gallic acid as the standard. The chlorogenic acid content was determined using UV-Vis spectrophotometry. The chlorogenic acid standard solution was prepared by accurately weighing 0.011 g of chlorogenic acid standard and dissolving it in 70% ethanol to a final volume of 100 mL, resulting in a 0.11 mg/mL solution. The maximum absorption wavelength was determined to be 331 nm. A standard curve was established by measuring the absorbance of chlorogenic acid solutions at different concentrations (1.0 mL, 1.5 mL, 2.0 mL, 2.5 mL, and 3.0 mL) at 331 nm. The chlorogenic acid content in the chrysanthemum extracts was calculated using the standard curve equation.

### 2.8. Untargeted Metabolomic Analysis Based on LC-MS/MS

Initially, each powdered sample was accurately weighed (50 mg) and placed into a 2 mL centrifuge tube. Subsequently, 600 µL of methanol (pre-stored at −20 °C) containing 2-chloro-L-phenylalanine (4 ppm) was added as the internal standard, followed by vortexing for 30 s to ensure thorough mixing. To facilitate sample homogenization, 100 mg of glass beads was introduced, and the sample was ground using a tissue grinder at a frequency of 60 Hz for 90 s. The sample was then sonicated at room temperature for 15 min to further disrupt cellular structures. Centrifugation was performed at 12,000 rpm and 4 °C for 10 min to separate the supernatant from the particulate matter. The supernatant was filtered through a 0.22 µm membrane and transferred into a vial for subsequent LC-MS analysis. The experiments were conducted in triplicate for biological replicates.

For liquid chromatography analysis, a Vanquish UHPLC System (Thermo Fisher Scientific, Waltham, MA, USA), equipped with an ACQUITY UPLC^®^ HSS T3 column (150 × 2.1 mm, 1.8 µm) (Waters, Milford, MA, USA), maintained at a constant temperature of 40 °C, was employed. The flow rate was set to 0.25 mL/min, with an injection volume of 2 μL. For LC-ESI (+)-MS analysis, the mobile phases consisted of 0.1% formic acid in acetonitrile (B2) and 0.1% formic acid in water (A2), following a gradient elution program: 0–1 min, 2% B2; 1–9 min, 2–50% B2; 9–12 min, 50–98% B2; 12–13.5 min, 98% B2; 13.5–14 min, 98–2% B2; and 14–20 min, 2% B2. For LC-ESI (-)-MS analysis, acetonitrile (B3) and 5 mM ammonium formate (A3) were used as mobile phases, with a similar gradient program: 0–1 min, 2% B3; 1–9 min, 2–50% B3; 9–12 min, 50–98% B3; 12–13.5 min, 98% B3; 13.5–14 min, 98–2% B3; and 14–17 min, 2% B3.

Mass spectrometric detection of metabolites was conducted using an Orbitrap Exploris 120 (Thermo Fisher Scientific, Waltham, MA, USA) equipped with an ESI ion source. Simultaneous acquisition of MS1 and MS/MS data (Full MS-ddMS2 mode, data-dependent MS/MS) was performed. The ESI(+) spray voltage was set to 3.50 kV, while the ESI(−) spray voltage was −2.50 kV. The capillary temperature was maintained at 325 °C, and the MS1 range was set to *m*/*z* 100–1000. The MS1 resolving power was 60,000 FWHM, with 4 data-dependent scans per cycle. The MS/MS resolving power was 15,000 FWHM, and a normalized collision energy of 30% was applied. Additional parameters included a sheath gas pressure of 30 arb, an auxiliary gas flow of 10 arb, and automatic dynamic exclusion time.

The raw mass spectrometry data were converted to mzXML format using the MSConvert tool within the ProteoWizard software package (v3.0.8789) [39]. Peak detection, filtering, and alignment were performed using the XCMS package [40] in R, with the following parameters: bw = 2, ppm = 15, peakwidth = c(5, 30), mzwid = 0.015, mzdiff = 0.01, and method = ‘centWave’. Compounds were identified using public databases such as HMDB [41], MassBank [42], LipidMaps [43], mzCloud [44], KEGG [45], and an in-house database, with a tolerance threshold of less than 30 ppm. To eliminate systematic errors, the LOESS signal correction method was applied to quality control (QC) samples. During data quality control, compounds with a relative standard deviation (RSD) greater than 30% in the QC samples were excluded. Statistical tests were conducted to calculate *p*-values, and orthogonal projections to latent structures-discriminant analysis (OPLS-DA) was used to determine variable importance in projection (*VIP*) scores and fold changes (FC), thereby evaluating the impact and explanatory power of each metabolite on sample classification. Metabolites with *p* < 0.05 and *VIP* > 1 were considered statistically significant differential accumulated metabolites (DAMs).

### 2.9. Data Statistical Analysis

Principal component analysis (PCA) and orthogonal partial least squares discriminant analysis (OPLS-DA) were performed using the R package “Ropls” [46]. Hierarchical clustering heatmap analysis was conducted using the complexHeatmap package [47]. One-way ANOVA, Spearman, and Pearson correlation analyses were performed using built-in R functions. Significant correlation networks were visualized using Cytoscape 3.10.1 [48].

## 3. Results

### 3.1. Comparative Analysis of Antioxidant and Sunscreen Properties of Extracts from Five Chrysanthemum Cultivars

**Figure 2:**
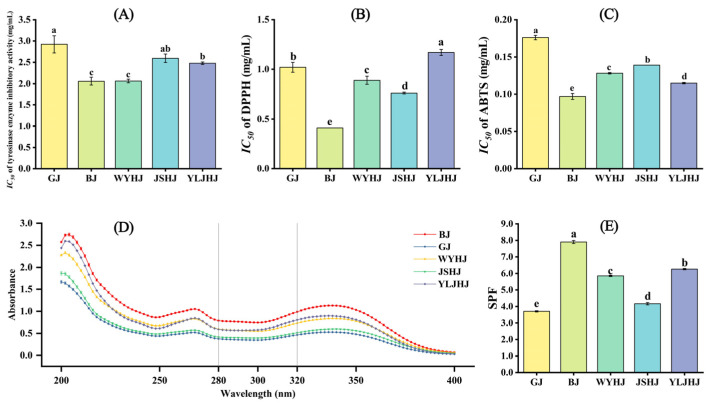
Evaluation of the antioxidant and whitening effects of extracts from five chrysanthemum varieties. (**A**) *IC_50_* of tyrosinase inhibitory activity; (**B**) *IC_50_* of DPPH radical scavenging rate; (**C**) *IC_50_* of ABTS radical scavenging rate; (**D**) ultraviolet absorption of the five varieties; (**E**) *SPF* value. Bar graphs or points and error bars represent mean ± S.E. (*n* = 3). Different lowercase letters indicate statistically significant differences at the *p* < 0.05 level.

In terms of tyrosinase inhibitory activity, the *IC_50_* values for GJ, BJ, WYHJ, JSHJ, and YLJHJ were 2.922 mg/mL, 2.057 mg/mL, 2.062 mg/mL, 2.596 mg/mL, and 2.479 mg/mL, respectively (Figure 2A and Figure S1). Lower *IC_50_* values indicate higher inhibitory activity. BJ and WYHJ exhibited the strongest tyrosinase inhibitory activity, with the lowest *IC_50_* values, followed by YLJHJ, while GJ had the highest *IC_50_* value. For DPPH radical scavenging activity, BJ had the lowest *IC_50_* value of 0.412 mg/mL, followed by JSHJ with an *IC_50_* of 0.764 mg/mL, and YLJHJ had the highest *IC_50_* of 1.165 mg/mL (Figure 2B and Figure S2). Regarding ABTS radical scavenging activity, BJ had the lowest *IC_50_* of 0.097 mg/mL, followed by YLJHJ, WYHJ, JSHJ, and GJ with *IC_50_* values of 0.115 mg/mL, 0.128 mg/mL, 0.139 mg/mL, and 0.176 mg/mL, respectively (Figure 2C and Figure S3).

Sunscreen performance was evaluated by measuring the absorbance of the chrysanthemum extracts in the UVA, UVB, and UVC ranges (200–400 nm). As shown in Figure 2D, all five chrysanthemum extracts exhibited good absorption across the UV spectrum, with varying intensities. BJ showed the strongest UV absorption across the entire spectrum, followed by YLJHJ and WYHJ, which had comparable sunscreen performance. In contrast, JSHJ and GJ had weaker UV absorption. Higher *SPF* values indicate stronger UVB protection. Further analysis of their *SPF* values revealed significant differences among the cultivars, with BJ having the highest *SPF* value, followed by YLJHJ and WYHJ, while JSHJ and GJ had lower *SPF* values (Figure 2E).

### 3.2. Comparative Analysis of Total Flavonoid, Phenolic, and Chlorogenic Acid Contents in Extracts from Five Chrysanthemum Cultivars

**Figure 3:**
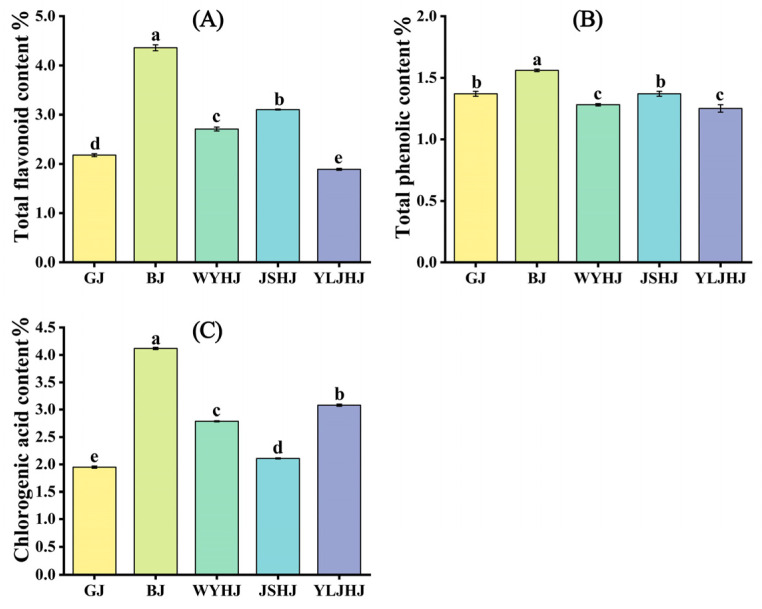
Comparison of total flavonoid content (**A**), phenolic content (**B**), and chlorogenic acid content (**C**) in five chrysanthemum cultivars. Bar graphs and error bars represent mean ± S.E. (*n* = 3). Different lowercase letters indicate statistically significant differences at the *p* < 0.05 level.

To understand the differences in active compound content among the five chrysanthemum cultivars, we measured their total flavonoid, phenolic, and chlorogenic acid contents. As shown in Figure 3, the content of these three active compounds varied significantly among the different cultivars. In terms of total flavonoid content, BJ had the highest content (4.36%), followed by JSHJ (3.10%), WYHJ (2.71%), and GJ (2.18%), with YLJHJ having the lowest content (3.08%) (Figure 3A). Regarding total phenolic content, BJ also had the highest content (1.56%), followed by GJ (1.37%) and JSHJ (1.37%), while WYHJ (1.28%) and YLJHJ (1.25%) had lower contents (Figure 3B). As for chlorogenic acid content, BJ had the highest content (4.12%), followed by YLJHJ (3.08%), WYHJ (2.79%), and JSHJ (2.11%), with GJ having the lowest content (1.95%) (Figure 3C).

### 3.3. Correlation Analysis of Three Compound Classes with Whitening Efficacy Indicators

**Figure 4:**
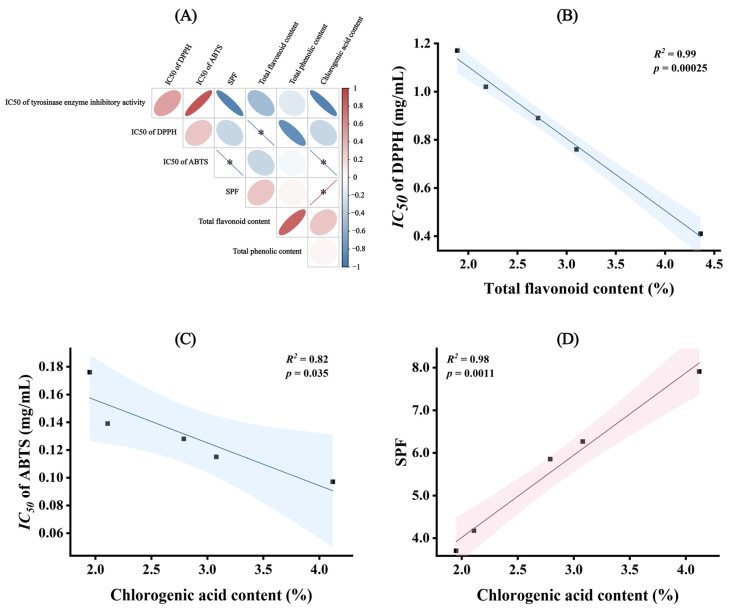
Correlation analysis of physiological indicators with antioxidant and whitening indicators. (**A**) Correlation heatmap; “*” indicates *p* < 0.05. Red indicates positive correlations, while blue indicates negative correlations. (**B**) General linear regression result between total flavonoid content and *IC_50_* of DPPH; (**C**) general linear regression result between chlorogenic acid content and *IC_50_* of ABTS; (**D**) general linear regression result between chlorogenic acid content and *SPF*. In (**B**–**D**), the black dots represent the numerical points corresponding to the two variables. The straight line depicts the fitted line from the general linear regression analysis. The shaded areas indicate the 95% confidence intervals.

To further investigate the active components influencing the whitening efficacy of the chrysanthemum extracts, we analyzed the correlation between the *IC_50_* values for tyrosinase inhibition, antioxidant activities (DPPH and ABTS), UV absorption capacity, *SPF* values, and the contents of flavonoids, total phenolics, and chlorogenic acid in the different chrysanthemum cultivars (Figure 4A). The results showed a significant negative correlation between total flavonoid content and the *IC_50_* of DPPH (*R^2^* = 0.99) (Figure 4B), a significant negative correlation between chlorogenic acid content and the *IC_50_* of ABTS (*R^2^* = 0.82) (Figure 4C), and a significant positive correlation between chlorogenic acid content and *SPF* values (*R^2^* = 0.98) (Figure 4D). This further emphasizes the roles of total flavonoids and chlorogenic acid in antioxidant and sunscreen activities.

### 3.4. Untargeted Metabolomic Analysis of Chrysanthemum Flower Extracts from Three Cultivars Based on LC-MS

**Figure 5:**
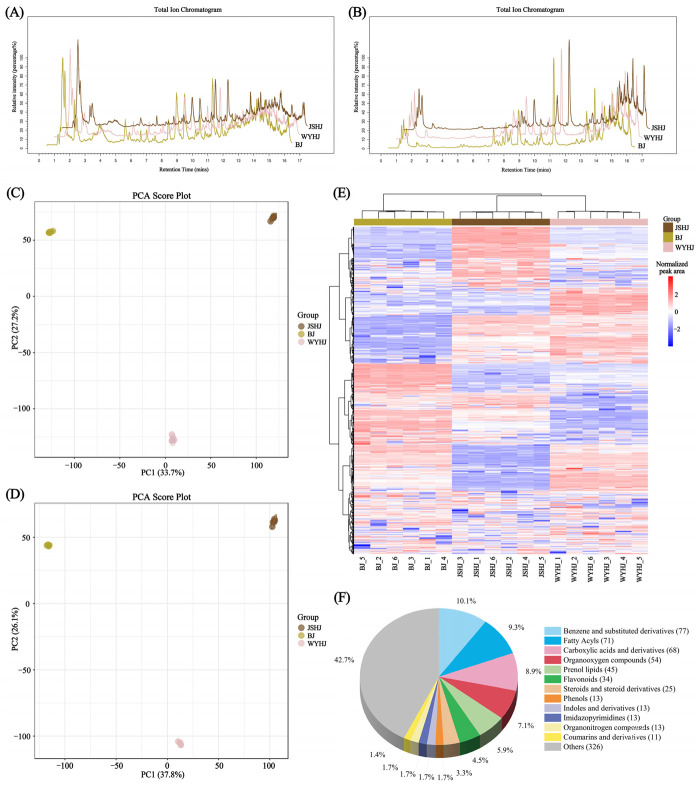
LC-MS/MS analysis of three representative chrysanthemum cultivars. (**A**) Base peak chromatograms in positive ion mode; (**B**) base peak chromatograms in negative ion mode; (**C**) PCA analysis in positive ion mode; (**D**) PCA analysis in negative ion mode; (**F**) classification of compounds; (**E**) hierarchical clustering heatmap analysis. In the heatmap, red indicates a larger peak area, while blue indicates a smaller peak area.

LC-MS scans were performed in both positive and negative ion modes. Figure 5A,B show the base peak chromatograms (BPC) of three representative chrysanthemum samples. Principal component analysis (PCA) results showed good within-group clustering and significant between-group dispersion. In the positive ion mode, the first principal component (PC1) explained 33.7% of the variance, and the second principal component (PC2) explained 27.2% (Figure 5C). In the negative ion mode, PC1 explained 37.8% of the variance, and PC2 explained 26.1% (Figure 5D). The HCA result distinctly demonstrates that replicates of the three chrysanthemum varieties cluster together within their respective categories, with clear delineations observed amongst the replicates, thereby indicating the existence of substantial metabolic variations amongst the chrysanthemum samples (Figure 5E).

**Figure 6:**
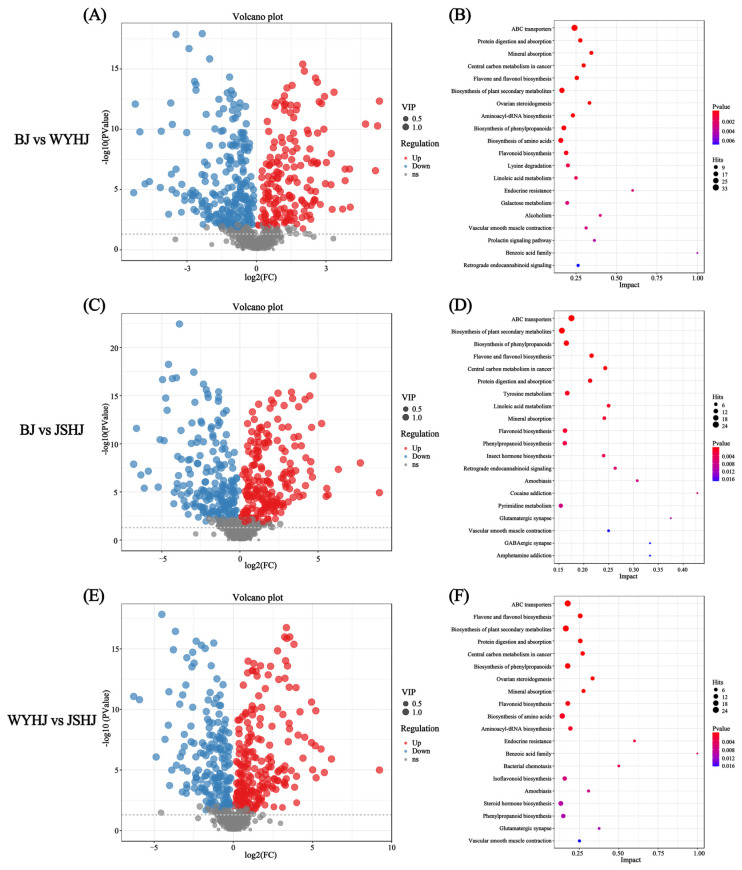
Differential metabolite analysis. (**A**) Volcano plot of DAMs between BJ and WYHJ. (**B**) KEGG enrichment analysis of DAMs between BJ and WYHJ; (**C**) Volcano plot of DAMs between BJ and JSHJ; (**D**) KEGG enrichment analysis of DAMs between BJ and JSHJ; (**E**) Volcano plot DAMs between WYHJ and JSHJ; (**F**) KEGG enrichment analysis of DAMs between WYHJ and JSHJ. Each point on the volcano plot represents a metabolite. Blue indicates significantly downregulated metabolites, while red indicates upregulated metabolites.

Using MS/MS fragmentation patterns, 763 differential metabolites were identified across the three chrysanthemum cultivars, including 68 carboxylic acids and derivatives, 34 flavonoids, 13 phenolic compounds, 11 coumarins and derivatives, 8 cinnamic acids and derivatives, 7 isoflavonoids, 5 quinolines and derivatives, and 3 alcohols and polyols (Figure 5F). In the comparison between BJ and WYHJ, 410 DAMs were identified, with 169 upregulated and 241 downregulated (Figure 6A). In the comparison between BJ and JSHJ, 397 differential metabolites were identified, with 205 upregulated and 192 downregulated (Figure 6C). In the comparison between WYHJ and JSHJ, 395 differential metabolites were identified, with 216 upregulated and 179 downregulated (Figure 6E).

To determine the metabolic pathways involved in the differential metabolites among the chrysanthemum cultivars, KEGG pathway enrichment analysis was performed, covering 238 metabolic pathways. Among them, the biosynthesis pathways of phenylpropanoids, flavone and flavonol, plant secondary metabolites, flavonoids, isoflavonoids, and amino acids have received particular attention due to their excellent antioxidant, anti-inflammatory, or whitening effects.

Analysis of the top 20 significantly enriched pathways for BJ vs. WYHJ revealed that these included pathways such as flavone and flavonol biosynthesis, biosynthesis of plant secondary metabolites, biosynthesis of phenylpropanoids, biosynthesis of amino acids, and flavonoid biosynthesis, encompassing 13, 23, 18, 20, and 14 differentially accumulated metabolites (DAMs), respectively (Figure 6B). Natural antioxidants such as apigenin, luteolin 7-O-glucuronide, cosmosiin, kaempferol, astragalin, and apigenin 7-O-neohesperidoside were significantly upregulated in BJ compared to WYHJ, with apigenin 7-O-neohesperidoside showing a 3.83-fold increase.

Analysis of the top 20 significantly enriched pathways for BJ vs. JSHJ identified 22 DAMs enriched in the biosynthesis of plant secondary metabolites pathway, 17 in the biosynthesis of phenylpropanoids pathway, 11 in the flavone and flavonol biosynthesis pathway, 13 in the tyrosine metabolism pathway, and 12 in the flavonoid biosynthesis pathway (Figure 6D). These pathways are related to metabolism, with the final products being the active ingredients of medicinal plants, indicating their collective influence on the physiological relevance of BJ and JSHJ. The flavone and flavonol biosynthesis and flavonoid biosynthesis pathways are interconnected, with many metabolites exhibiting antioxidant properties.

Analysis of the top 20 significantly enriched pathways for WYHJ vs. JSHJ revealed 13 DAMs in the flavone and flavonol biosynthesis pathway, 23 in the biosynthesis of plant secondary metabolites pathway, 18 in the biosynthesis of phenylpropanoids pathway, 13 in the flavonoid biosynthesis pathway, 18 in the biosynthesis of amino acids pathway, and 10 in the isoflavonoid biosynthesis pathway (Figure 6F).

In the top 20 significantly enriched pathways across all three comparisons, pathways such as flavone and flavonol biosynthesis, flavonoid biosynthesis, biosynthesis of plant secondary metabolites, and biosynthesis of phenylpropanoids were consistently included. This underscores their potential roles in the differences in antioxidant and whitening effects among the extracts of different chrysanthemum varieties.

### 3.5. K-Means Analysis and Identification of Key Compounds for Sunscreen Efficacy

**Figure 7:**
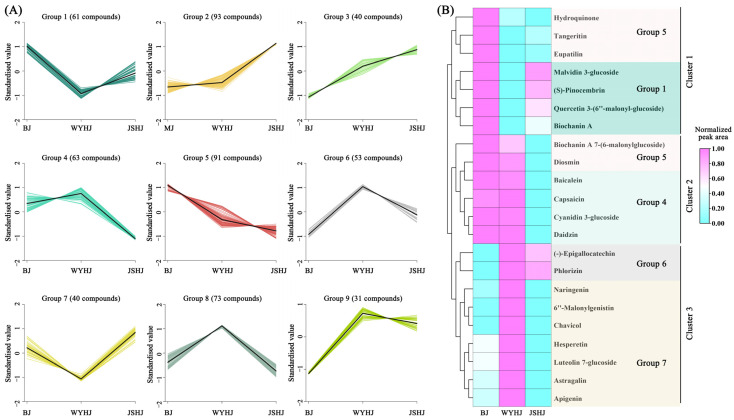
K-means analysis of differential metabolites (**A**) and hierarchical clustering analysis of active compounds (**B**). In Figure (**A**), the black line represents the trend of the relative content of the compound across the extracts of three varieties, while the colored lines depict the relative content of various compounds within different groups across the extracts of the same three varieties. In the heatmap, pink indicates a larger peak area, while light blue indicates a smaller peak area.

To identify key compounds affecting the antioxidant and sunscreen efficacy of the different chrysanthemum cultivars, k-means analysis was performed on 545 DAMs, resulting in nine groups. Groups 1 to 9 contained 61, 93, 40, 63, 91, 53, 40, 73, and 31 compounds, respectively (Figure 7A). Based on the antioxidant and sunscreen efficacy analysis, groups with higher relative compound content in BJ and WYHJ were prioritized. Compounds in Groups 1 and 5 had the highest content in BJ, while compounds in Group 4 had high content in both BJ and WYHJ. Compounds in Groups 6 and 8 had the highest content in WYHJ. A total of 22 compounds with antioxidant or whitening sunscreen efficacy were identified in Groups 1, 4, 5, 6, and 7, including 15 flavonoids (hesperetin, astragalin, cyanidin 3-glucoside, tangeritin, malvidin 3-glucoside, diosmin, (S)-pinocembrin, (-)-epigallocatechin, quercetin 3-(6″-malonyl-glucoside), baicalein, apigenin, naringenin, phlorizin, luteolin 7-glucoside, eupatilin), 4 isoflavonoids (biochanin A, 7-(6-malonylglucoside), 6″-malonylgenistin, biochanin A, daidzin), and 3 phenols (capsaicin, chavicol, hydroquinone). HCA of these 22 compounds revealed three clusters, which largely corroborated the k-means analysis results (Figure 7B; Table S1). Cluster 1 included three compounds from Group 5 and four from Group 1, with high content in BJ and low content in JSHJ and WYHJ. Cluster 2 included two compounds from Group 5 and four from Group 4, with high content in BJ and WYHJ and low content in JSHJ. Cluster 3 included two compounds from Group 6 and seven from Group 7, with high content in WYHJ.

### 3.6. Correlation Analysis of Key Bioactive Compounds with Antioxidant and Sunscreen Indicators

**Figure 8:**
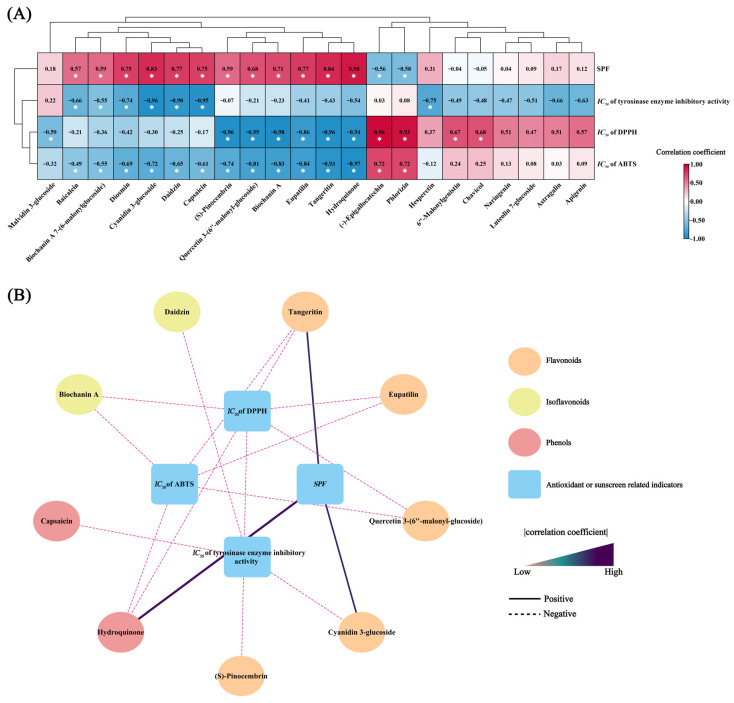
Identification of key compounds related to antioxidant and whitening effects. (**A**) Correlation heatmap; “*” Indicating *p* < 0.05. Red blocks represent positive correlations, while blue blocks represent negative correlations. (**B**) Significant correlation network visualization (*p* < 0.05, |correlation coefficient| > 0.8). Solid lines and purple-black color indicate positive correlations, whereas dashed lines and pink color indicate negative correlations.

To further identify key compounds related to antioxidant and sunscreen indicators, we performed Spearman correlation analysis on the 22 compounds identified in Section 3.5 with *SPF*, *IC_50_* of DPPH, *IC_50_* of ABTS, and *IC_50_* of tyrosinase. The results showed that 12 compounds were significantly positively correlated with *SPF*; 7 compounds were negatively correlated with *IC_50_* of tyrosinase enzyme inhibitory activity; 7 compounds were significantly negatively correlated with *IC_50_* of DPPH; and 12 compounds were significantly positively correlated with *IC_50_* of ABTS (Figure 8A).

A correlation network was constructed with *p* < 0.05 and |*r*| > 0.8 (Figure 7B and Table S2). The network revealed that three compounds were significantly positively correlated with *SPF*, including two flavonoids (tangeritin and cyanidin 3-glucoside) and one phenol (hydroquinone); one flavonoid (cyanidin 3-glucoside), one isoflavonoid (daidzin), and one phenol (capsaicin) were significantly negatively correlated with *IC_50_* of tyrosinase enzyme inhibitory activity; four flavonoids (tangeritin, eupatilin, quercetin 3-(6″-malonyl-glucoside), and (S)-pinocembrin), one isoflavonoid (biochanin A), and one phenol (hydroquinone) were significantly negatively correlated with *IC_50_* of DPPH; and three flavonoids (tangeritin, eupatilin, quercetin 3-(6″-malonyl-glucoside)), one isoflavonoid (biochanin A), and one phenol (hydroquinone) were significantly negatively correlated with *IC_50_* of ABTS. Some compounds exhibited multiple antioxidant or sunscreen effects, with tangeritin and hydroquinone significantly correlated with *SPF*, *IC_50_* of DPPH, and *IC_50_* of ABTS. Additionally, eupatilin, quercetin 3-(6″-malonyl-glucoside), and biochanin were significantly negatively correlated with both *IC_50_* of DPPH and *IC_50_* of ABTS. Cyanidin 3-glucoside was also significantly correlated with both *SPF* and *IC_50_* of tyrosinase enzyme inhibitory activity, highlighting the importance of these compounds in antioxidant and whitening effects.

## 4. Discussion

Previous studies have shown that chrysanthemum essential oils [21], teas [12,49], aqueous extracts [50], and organic extracts [24] possess significant antioxidant and anti-inflammatory properties. In this study, ethanol extracts of chrysanthemum obtained through vacuum freeze-drying were evaluated for their antioxidant and whitening effects. The results indicated significant differences in the *IC_50_* values for tyrosinase enzyme inhibitory activity, DPPH, ABTS, UV absorption rates, and *SPF* values among the five chrysanthemum cultivars. The extracts from different chrysanthemum genotypes exhibited notable differences [21,51], highlighting the necessity of evaluating the efficacy of extracts from various cultivars. Among the five cultivars, BJ demonstrated the strongest antioxidant effect, tyrosinase inhibition, and UV absorption, and the highest *SPF* value. Previous studies have also reported that chrysanthemum extracts possess tyrosinase inhibitory activity [52] and good UV absorption [23], consistent with our findings. This suggests that chrysanthemum extracts, particularly from the BJ cultivar, have significant potential for application in whitening cosmetics.

Phenolics, flavonoids, and chlorogenic acid are important active compounds in chrysanthemum extracts [51]. Additionally, as natural compounds, they are widely used in cosmetics due to their antioxidant, anti-inflammatory, antiviral, and whitening effects [53]. This study also analyzed the total flavonoid, total phenolic, and chlorogenic acid contents in the five chrysanthemum cultivars, revealing significant differences among the genotypes. BJ had the highest levels of total flavonoids, total phenolics, and chlorogenic acid, correlating with its superior antioxidant, UV absorption, and whitening effects. Correlation analysis further revealed significant relationships between flavonoid content and *IC_50_* of DPPH, and between chlorogenic acid content and both *IC_50_* of ABTS and *SPF* values. A study also found that high levels of flavonoids in chrysanthemum extracts are associated with antioxidant activity [20]. Additionally, phenolic compounds in chrysanthemum extracts have been found to correlate strongly with antioxidant indicators [54]. Although our study found a high correlation coefficient between total phenolic content and *IC_50_* of DPPH, it was not significant, possibly due to the number of cultivars studied and the total phenolic content extracted.

This study conducted untargeted metabolomic analysis on the petals of three representative chrysanthemum cultivars and identified 763 compounds, including recognized bioactive components such as flavonoids (34 types) and phenolics (13 types), which align well with previous research on other chrysanthemum varieties [12,14]. KEGG enrichment analysis revealed that many differentially accumulated metabolites (DAMs) in the three comparisons were significantly enriched in the biosynthesis pathways of phenylpropanoids, flavones and flavonols, plant secondary metabolites, and flavonoids. These findings highlight that DAMs in these metabolic pathways may play a crucial role in the differences in antioxidant and whitening effects among the extracts of the three cultivars. Further correlation analysis identified 11 compounds significantly associated with antioxidant and sunscreen indicators, including tangeritin and hydroquinone, which showed significant correlations with *SPF*, *IC_50_* of DPPH, and *IC_50_* of ABTS, indicating their potential whitening efficacy. Tangeritin has been shown to have important antioxidant, anti-inflammatory, and neuroprotective effects [55,56]. Hydroquinone is known for its gold-standard skin whitening efficacy, corroborating our findings [57]. However, it has been reported to have cytotoxic effects on the skin [57] and may cause inflammation [58]. As a result, it has been recently banned [3]. This has led to widespread interest in natural plant extracts, which are considered safer alternatives to pure hydroquinone as whitening agents. In this study, hydroquinone was identified in natural extracts, which may offer better safety compared to its pure form. Because the hydroquinone content in natural extracts is relatively low and differs in form from pure hydroquinone, it may interact with other antioxidants in the extracts, though further evaluation is needed. As a traditional herbal medicine, chrysanthemum extracts have long been used to promote skin health and treat skin diseases [59]. Therefore, compared to pure hydroquinone, chrysanthemum extracts may serve as a safer alternative, but further clinical trials are necessary. Additionally, eupatilin [60], quercetin 3-(6″-malonyl-glucoside) [60], biochanin A [61], and cyanidin 3-glucoside [62], identified in this study, have been reported to possess strong antioxidant properties. Therefore, despite the safety concerns associated with hydroquinone, our study demonstrates the potential value of these natural extracts in whitening cosmetic formulations. Furthermore, daidzin (isoflavonoid), capsaicin (phenol), and cyanidin 3-glucoside (flavonoid) show a significant correlation with *IC_50_* of tyrosinase inhibitory activity. Since daidzin [63], capsaicin [64], and cyanidin 3-glucoside [65] have been reported as bioactive components with tyrosinase inhibitory activity, we speculate that these three components are key contributors to the tyrosinase inhibitory activity of chrysanthemum extracts in this study. However, the mechanism of tyrosinase inhibition by the combined chrysanthemum extracts requires further exploration.

Significant differences in the antioxidant and whitening effects of chrysanthemum extracts were observed across various genotypes. For instance, a purple chrysanthemum and its radiation-induced mutant tea showed noticeable differences in antioxidant activity [12]. Essential oils from different chrysanthemum species, such as *Dendranthema morifolium*, *Pericallis hybrida*, and *Bellis perennis*, also exhibited significant differences in DPPH and FRAP C (Fe^2+^) radical scavenging rates [21]. Our study also found that ‘Boju’ (BJ), one of China’s four famous chrysanthemums, had the strongest tyrosinase inhibitory activity, antioxidant activity, and *SPF* value among the five chrysanthemum varieties studied. These results underscore the importance of genotype screening and analysis when evaluating the antioxidant or whitening efficacy of chrysanthemum extracts. This highlights the importance of carefully selecting genotypes when developing natural extracts from other plants for use in cosmetics, as the bioactivity and efficacy of the extracts can vary significantly between different genotypes. This screening provides valuable references for selecting raw materials for industrialization among the numerous chrysanthemum varieties.

Future clinical trials on the whitening effects of chrysanthemum extracts are needed. Additionally, it should be clarified that the cultivars in this study were grown under specific cultivation conditions, and the methods for harvesting and processing the raw materials were also specific. These factors represent limitations of this study. Therefore, following the confirmation of clinical results, further optimization of post-harvest processing, raw material handling, and production processes is required.

## 5. Conclusions

This study highlights the significant variations in antioxidant and whitening properties among five chrysanthemum varieties from Guangdong Province, China. The BJ variety, in particular, demonstrated superior performance in both antioxidant capacity and whitening efficacy, attributed to its high levels of total flavonoids, total phenols, and chlorogenic acid. Correlation analysis revealed meaningful relationships between specific compounds and their bioactivities, suggesting that these phytochemicals play a crucial role in the observed effects. The non-targeted metabolomics approach identified 22 compounds potentially contributing to the antioxidant and whitening properties, underscoring the BJ variety’s potential as a valuable source of natural ingredients for cosmetic and health applications. The research results provide potential raw material options for developing safer and more effective natural whitening agents. Additionally, these findings lay the foundation for further exploration and utilization of chrysanthemum extracts in various industries.

## Figures and Tables

**Table 1:** The normalized product function used in the calculation of *SPF* data [36].

Wavelength λ (nm)	*EE* (λ) × *I* (λ) (Constant)
290	0.015
295	0.081
300	0.2874
305	0.3278
310	0.1864
315	0.0839
320	0.0180

## Data Availability

Data is contained within the manuscript and Supplementary Materials.

## Supplementary Materials

The following supporting information can be downloaded at https://www.mdpi.com/article/10.3390/antiox13121557/s1. Figure S1: Comparative analysis of tyrosinase inhibitory activity; Figure S2: Comparative evaluation of DPPH radical scavenging capacity; Figure S3: Comparative assessment of ABTS radical scavenging efficiency; Table S1: Grouping of DAMs in k-means clustering; Table S2: Results of correlation analysis between key compounds and antioxidant and skin-whitening efficacy (*p* < 0.05, |*r*| > 0.8).
