# Supplementary material for: Evaluation of Whitening Effects and Identification of Potentially Active Compounds Based on Untargeted Metabolomic Analysis in Different Chrysanthemum Cultivar Extracts
Source: Antioxidants (Basel). 2024 Dec 18;13(12):1557. doi: 10.3390/antiox13121557 (PMC11673076; doi:10.3390/antiox13121557)
Supplement: Supplementary file 1 [file antioxidants-13-01557-s001.zip › Supplementary materials/Supplementary Figures.pdf]

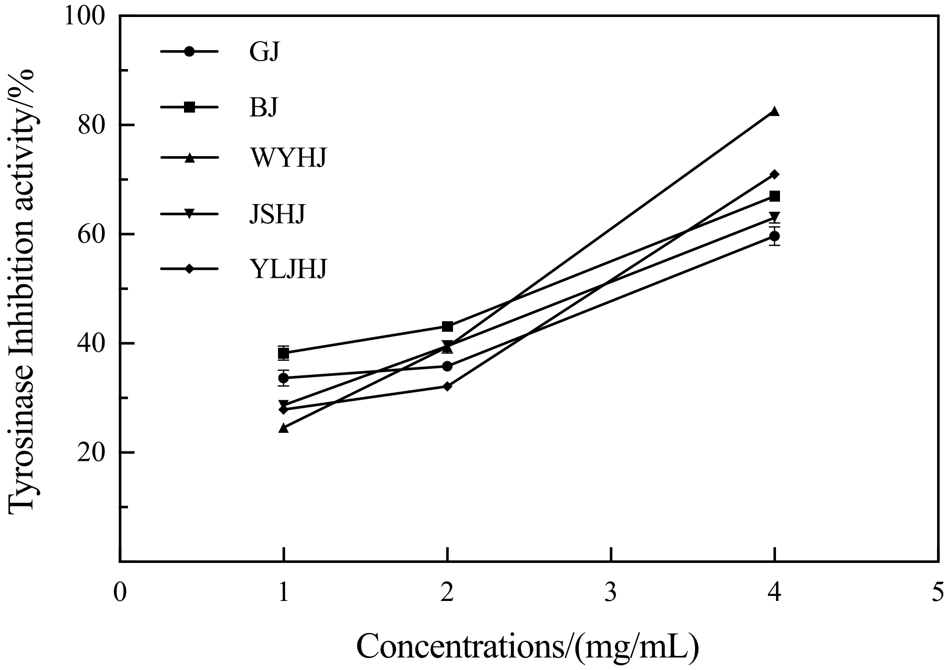

Figure S1. Comparative analysis of tyrosinase inhibitory activity.

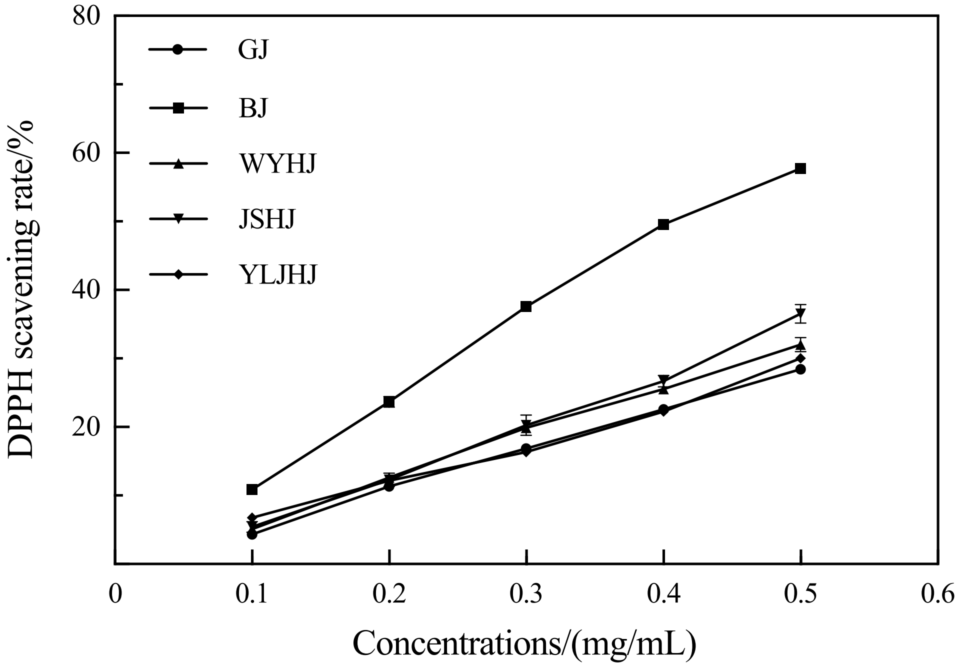

Figure S2. Comparative evaluation of DPPH radical scavenging capacity.

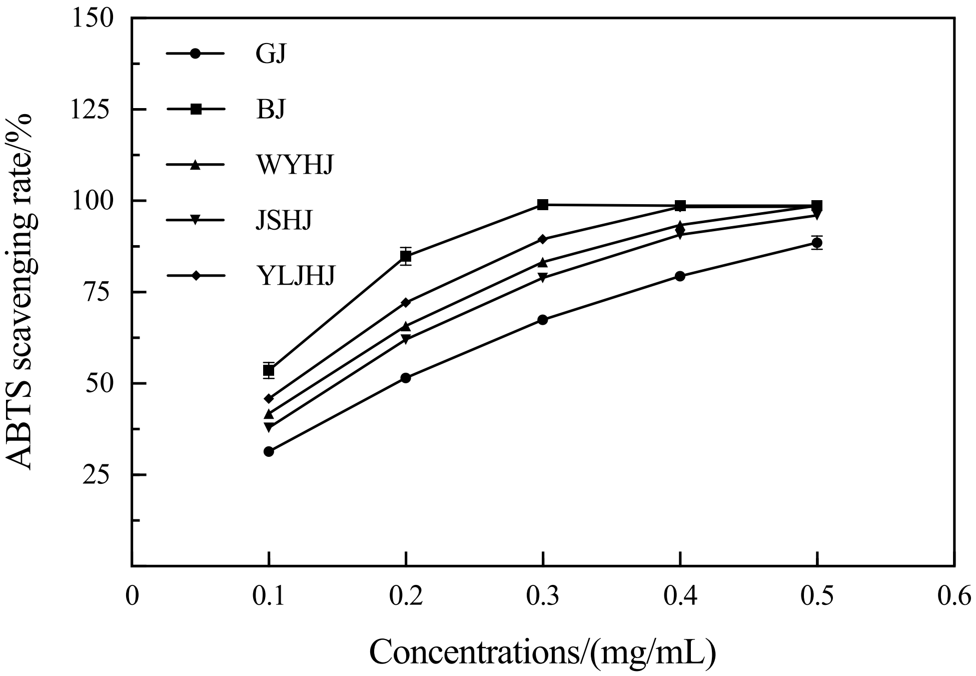

Figure S3. Comparative evaluation of ABTS radical scavenging capacity.
